# Cancer-Associated Fibroblasts and Extracellular Matrix: Therapeutical Strategies for Modulating the Cholangiocarcinoma Microenvironment

**DOI:** 10.3390/curroncol30040319

**Published:** 2023-04-14

**Authors:** Mirko Minini, Laura Fouassier

**Affiliations:** 1 Centre de Recherche Saint-Antoine, CRSA, Sorbonne Université, INSERM, 75012 Paris, France; 2 Association Pour L’étude des Cancers et Affections des Voies Biliaires (ACABi), 75012 Paris, France

**Keywords:** cancer-associated fibroblasts, extracellular matrix, immune-exclusion, tumor microenvironment

## Abstract

During the last decade, immunotherapy has radically changed perspectives on anti-tumor treatments. However, solid tumor treatment by immunotherapy has not met expectations. Indeed, poor clinical response to treatment has highlighted the need to understand and avoid immunotherapy resistance. Cholangiocarcinoma (CCA) is the second cause of hepatic cancer-related deaths because of drug inefficacy and chemo-resistance in a majority of patients. Thus, intense research is ongoing to better understand the mechanisms involved in the chemo-resistance processes. The tumor microenvironment (TME) may be involved in tumor therapy resistance by limiting drug access. Indeed, cells such as cancer-associated fibroblasts (CAFs) alter TME by producing in excess an aberrant extracellular matrix (ECM). Interestingly, CAFs are the dominant stromal component in CCA that secrete large amounts of stiff ECM. Stiff ECM could contribute to immune exclusion by limiting anti-tumor T-cells drop-in. Herein, we summarize features, functions, and interactions among CAFs, tumor-associated ECM, and immune cells in TME. Moreover, we discuss the strategies targeting CAFs and the remodeling of the ECM to improve immunotherapy and drug therapies.

## 1. Introduction

Cholangiocarcinoma [1], a biliary tract cancer, is the second most common cause of hepatic cancer-related deaths. CCA is characterized by an increasing annual incidence and an unsatisfactory treatment outcome. Indeed, a considerable fraction of patients under systemic chemotherapy for advanced unresectable CCA fails to respond to treatment [2], leading to a poor prognosis with a five-year overall survival rate of less than 30% [3,4]. Thus, a better understanding of the cellular and molecular mechanisms leading to drug resistance is urgently needed to develop more effective treatments against CCA.

The tumor microenvironment (TME) plays a central role in tumor development and progression. During tumor progression, TME undergoes profound changes that give rise to increased extracellular matrix (ECM) deposition and altered tissue architecture [5]. The latter changes may elicit modifications leading to a resistant tumor status [6,7]. Cancer-associated fibroblasts (CAFs), the main stromal cell types within the TME [8,9,10], play a key role in secreting multiple cytokines, chemokines, and soluble factors. CAFs thus have a role in modifying TME and controlling drug access to the tumor [11,12,13]. Notably, CCA presents itself with a high amount of ECM and number of CAFs and is thus the prototype of a tumor containing a stiff desmoplastic stroma [2,14].

In recent years, interest has grown regarding the role of the ECM and CAFs in TME and tumor development. High throughput technologies and mechano-immunology have allowed deciphering the role of CAFs and their secreted ECM in modulating the immune system in several tumors. The features of tumor-associated immune cells and ECM are more and more thoroughly investigated for their implications regarding drug resistance, immunotherapy resistance, and anti-tumoral immunity. Indeed, biochemical interactions between some ECM components and immune cells, such as T-cells, macrophages, and myeloid-derived suppressor cells (MDSCs), can occur via specific cell surface receptors, such as CD44, RHAMM (CD168) [15], and DDR1 [16]. Therefore, the increased deposition of an altered ECM can offer physical and biochemical protection against immune surveillance.

The impact of CAFs on tumor progression, drug delivery, and effectiveness has been linked to the production of large amounts of altered ECM [17]. Furthermore, CAFs modulate liver stiffness [18,19] by dysregulating collagen turnover [20] and its cross-linking in the extracellular space. The spilling of ECM components in the extracellular space provides migration cues to tumor cells [21,22]. Once a desmoplastic microenvironment is established by CAFs, epithelial tumor cells can contribute to matrix deposition at later stages of tumor progression [23]. In CCA, IGF2/IGFR1/IR CAFs-activated axis was recently outlined by our team as a pathway involved in tumor cell plasticity, desmoplastic reaction, and resistance to anti-EGFR therapy [11]. Modification of TME in CCA is further supported by the evaluation of stiffness by shear wave elastography [24] that indicates that CCA has a stiffness (57 ± 25 kPa) statistically higher than hepatocellular carcinoma (15 ± 10 kPa) and tumor-free liver parenchyma (2.5 ± 2.5 kPa). Interestingly, CCA stiffness increases with tumor progression [25,26,27]. CCA tissue shows high levels of collagen fibers (i.e., COL3A1) [28] and a high collagen reticulation index (CRI), defined as the number of collagen fiber branches within the entire length of the collagen network. Levels of collagen fibers and high CRI are associated with poorer overall survival of CCA patients [29]. The concept aligns with a recent work showing tumor-associated collagen signatures (TACS) as significant predictors of tumor progression and therapy resistance [30].

Herein, we summarize the features, functions, and interactions of CAFs, tumor-associated ECM, and immune cells in TME. Moreover, we discuss the therapeutic strategies based on targeting and remodeling CAFs and ECM for improving immunotherapy and others tumor therapies.

## 2. Cancer-Associated Fibroblasts in the Tumor Microenvironment

### 2.1. CAF Origin

In normal tissues, fibroblasts can be defined as the cell type mainly involved in maintaining tissue homeostasis. The secretion and deposition of a tissue-specific ECM support tissue architecture and bioavailability of chemical factors in the microenvironment. However, fibroblasts and tissue homeostasis are altered in a pathological condition, such as tumor disease. Within the TME, fibroblast and mesenchymal-like cells result in activating CAFs that display a myofibroblastic phenotype. Specifically, these activated cells can arise from local fibroblasts, pericytes, hematopoietic stem cells, adipocytes, and epithelial and endothelial cells. In addition, CAFs can develop from bone marrow-derived mesenchymal stem cells (MSCs) recruited to the tumor site for tissue repair [31].

The fibroblast-like cell populations in the hepatic and pancreatic tissues are mainly represented by hepatic stellate cells (HSCs) and pancreatic stellate cells (PSCs). In the tumor context, these populations are another critical source of CAFs, becoming activated and assuming specific features [32,33].

Given the considerable origin heterogeneity, different CAF subtypes could be present within the TME. The evaluation and combination of several biomarker expressions and morphological features have allowed the identification, definition, and isolation of CAFs. Specifically, the CAF population isolated from tumor tissue should be negative for the expression of non-mesenchymal biomarkers (such as EpCAM or CD45). Moreover, CAFs should display a positive expression for mesenchymal biomarkers, such as vimentin, alpha-smooth muscle actin (α-SMA), fibroblast activation protein (FAP), and platelet-derived growth factor alpha/beta (PDGFα/β) [34,35]. The expression of these specific CAF markers has been associated with poor clinical prognosis in various cancer types [31]. That is why in recent years, several researchers have focused on the mesenchymal cells within the TME. In this context, novel high-throughput technologies (such as single-cell RNA sequencing (scRNA-seq), multicolor flow cytometry, and multiplex immunofluorescence) have enhanced the definition of biomarkers panels broadly recognized as expressed by CAFs.

Therefore, a better understanding of the CAFs expression profile could help the identification of specific subtypes enriched in different tumors and open new therapeutic frontiers.

### 2.2. CAF Subtypes and Biomarkers

As previously described, CAFs are heterogeneous. The heterogeneity is also well reflected in spatial localization within the TME, partly explaining the tumor heterogeneity [36,37,38]. Different CAF subtypes have been identified in tumors from various origins in the last decade.

In PDAC, two spatially and functionally distinct CAF subsets have been identified and characterized [32]. A myofibroblastic CAF subtype (myCAF) was identified within the peri-glandular region in proximity to tumor foci. The myCAFs are αSMA^hi^ positive, express low levels of inflammatory mediators, and are functionally specialized in stromal remodeling functions. An inflammatory CAF subtype (iCAF) was also identified and localized distant from the tumor cells. The iCAF expresses αSMA^low^ and high levels of inflammatory mediators (i.e., IL-1, IL-6, IL-11, LIF, and CXCL-1). The appearance of one of the two functional phenotypes occurs by the alternative and balanced expression of IL-1 Receptor type 1 with TGFβ (pro-myCAF) or IL-1/JAK/STAT (pro-iCAF) signaling [39]. Moreover, scRNA-seq allowed the identification of an additional CAF subtype described as antigen-presenting CAFs (apCAFs). The apCAFs represent a specific subset expressing the major histocompatibility complex (MHC) class II family genes as well as CD74. The singular expression profile supports an immunomodulatory role of apCAFs enabling the activation of and interaction with CD4^+^ T-cells [40].

In breast (BC) and ovarian (OC) cancers [8,41], four different CAF subtypes (CAF-S1 to CAF-S4) have been identified. The subset of myCAF (CAF-S1, CD29^med^ FAP^hi^ FSP-1^low-hi^ αSMA^hi^ PDGFRβ^med-hi^ CAV-1^low^) was localized close to tumor foci and displayed stromal remodeling functions and inflammatory activities (CCL-11, CXCL-12, CXCL-13, CXCL-14) [8]. Another myCAF subset (CAF-S4, CD29^hi^ FAP^neg-low^ FSP-1^low-med^ αSMA^hi^ PDGFRβ^low-med^ CAV-1^low^) was located in tumor foci and characterized by a perivascular signature [8]. In aggressive breast cancers (triple negative and HER2) and metastatic lymph nodes, high levels of CAF-S1 and CAF-S4 subsets have been observed, highlighting a role in immune suppression and pro-tumoral effect [8]. Remarkably, CAF-S1 and CAF-S4 subsets and biomarkers were validated in several tumors [38,41,42,43]. The partial overlapping of the CAF signature within other tumors raises the possibility of matching features for shared CAF subtypes in similar tumors.

**Figure 1:**
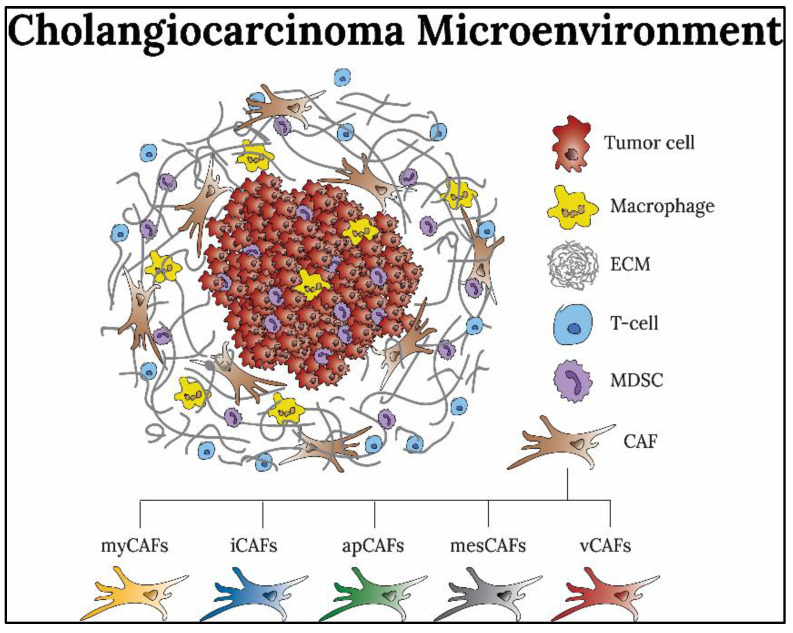
Overview of cholangiocarcinoma microenvironment and the main cancer-associated fibroblast subsets.

In CCA, the origin and functions of distinct CAF subsets promoting intrahepatic CCA growth were recently identified. While myCAF and iCAF subsets were identified as derived from HSCs, a novel identified rare subset, mesothelial CAF (mesCAF), originates from portal fibroblasts and expresses mesothelial markers (*Msln*, *Upk1b*, and *Upk3b*). Analysis of iCAF and myCAF subsets originating from CCA, by proteomic validation of scRNA-seq datasets, identified high expression of HGF and HAS2 (Hyaluronic Acid Synthase 2), respectively [33]. Another analysis by scRNA-seq revealed five distinct fibroblast subtypes (from 0 to 4) in human intrahepatic CCA, partially overlapped with myCAF, iCAF, apCAF, and mesCAF subsets. In addition, the tumor core and microvascular regions were found to be mainly infiltrated by vascular CAFs (vCAFs), a CD146^+^ population expressing IL-6/STAT3 signaling [44] (Figure 1 and Table 1). In another study, identifying distinct CAF subsets linked to different immune infiltration in human CCA has highlighted a novel possibility for patient stratification based on TME features. The four identified CCA subgroups are defined by a spectrum of immune and TME signatures from the immune desert (I1) to a slight immune infiltration (I2), up to the infiltration of myeloid (I3) and mesenchymal (I4) cells [45]. Through this classification, the therapeutic effectiveness and strategy are correlated and designed for the more complex TME, taking another step toward personalized medicine [46].

### 2.3. CAF Activated Signaling and Targeted Therapy

Therefore, CAF subsets are differently located within the TME. The localization is essential in establishing specific interactions and signals with the other cells in the TME. In addition to the paracrine signaling, many growth factors and cytokines secreted by CAFs are involved in maintaining and differentiating CAF subsets [47], together with a tumor-specific ECM [48]. Indeed, CAFs secretome is heterogeneous and may result from a balance in autocrine and paracrine signaling. Among them, an important role is undoubtedly associated with the TGFβ superfamily members known to induce a pro-tumorigenic phenotype in fibroblasts in several tumors [49,50]. Likewise, activin A is essential for the secretion of ECM components, regulators, and other soluble factors via SMAD2 signaling [51]. Therefore, the different signaling activated could be able to define the expression of various markers and phenotypical features of several CAF subtypes. Moreover, activating pathways involved in the secretion and deposition of ECM enhanced the remodeling of the TME.

Within the TME, the dynamic interactions between the different cell types and the ECM modulate and support tumor progression. In particular, these interactions limit the response to anti-tumor therapies [52] and provide a protective barrier against immunosurveillance together with the diffusion of anti-tumor factors [53]. Indeed, CAFs communicate with tumor-infiltrating immune cells to drive an immunosuppressive microenvironment [6,8,32]. CAFs also strongly contribute to generating and maintaining an immune-suppressed microenvironment by secreting WNT-2, a suppressor in differentiating and activating dendritic cells [54]. Furthermore, the drop-in of the anti-tumor T-cells and their contact with tumor cells is hampered by a stiff TME contributing to immune exclusion [16,55,56].

The targeted therapies aimed at remodeling the TME have shown promising results in CCA. Navitoclax, an inhibitor of Bcl-XL/Bcl-2 proteins, triggers cell death. However, tumor cells can develop resistance to navitoclax by increasing the expression of Mcl-1, an anti-apoptotic factor. Yet, observing that CAFs are sensitive to navitoclax has opened novel approaches for tumor therapy in CCA. Indeed, targeting CAFs by navitoclax could be sufficient to reduce the desmoplastic reaction, ECM deposition, and tumor growth [57]. In pancreatic cancer, depleting the CAF FAP^+^ population slowed tumor growth and increased CD8^+^ T-cells tumor infiltration [58]. However, clinical trials assessing the FAP-specific antibody (sibrotuzumab) in metastatic colorectal cancer were not conclusive, failing at phase II [24]. Furthermore, direct and selective depletion of the CAF αSMA+ population in pancreatic cancer resulted in poorly differentiated tumors, increased metastatic spreading and intra-tumoral immunosuppression, leading to decreased survival overall [59]. Taken together, the latter observations call for the advanced development of new immunotherapeutic approaches, particularly those addressing vaccination strategies or activation of the innate immune system [60] (Table 2).

## 3. Extracellular Matrix in the Tumor Microenvironment

### 3.1. ECM Composition and Targeting

Therefore, effective treatment and the normalization of the tumor stroma could lead to beneficial effects for cancer patients. Indeed, the high presence of CAFs in the TME strongly correlates with a poor patient prognosis in numerous cancers [61,62,63]. CAFs can organize and remodel the TME by expressing ECM proteins accounting for up to 60% of the tumor mass [64]. Secretion of tumor-associated ECM molecules leads to significant differences in amount, composition, and organization compared to the normal tissue’s ECM. The altered ECM is pointed out by the alternative splicing of genes coding ECM molecules. Observed alterations impact the establishment of tumor niche, angiogenesis, and immune response, enhancing tumor resistance [65].

The ECM has been found altered in several tumors in terms of components, deposition, and modification. Higher collagen deposition is one of the main features of desmoplastic TME, making the tumor stiffer than healthy tissue [66,67]. Collagen has several isoforms and is secreted as fibers. Tumor cells can remodel and organize these fibers by generating regions of highly aligned collagen fibers [68]. The alignment provides sticky rails for migrating tumor cells, reducing the energy required for the migration and facilitating intravasation [69,70]. Patterns of collagen isoform expression can be associated with poor prognosis in melanoma, breast, ovarian and lung cancers [71,72,73,74]. Consistently, the inhibition of CAF-mediated collagen I synthesis has shown a reduction of tumor growth in preclinical models [75,76] and phase II trials [77].

Like collagen, proteoglycans (PGs) and glycosaminoglycans (GAGs) are essential components of ECM. Hyaluronic acid (HA) is a GAG found to be increased in several tumors [78,79]. The reduction of CAF-derived HA deposition by PEGPH20, a hyaluronidase, resulted in favorable outcomes in a phase II trial [80], but the subsequent phase III was interrupted for lack of efficacy [81].

The expression profile and deposition of ECM significantly differ among tumors. Thus, the evaluation of an ECM signature represents a practical and predictive component for overall and disease-free patient survival [82]. Accordingly, an ECM-based diagnostic tool could help the patient’s stratification [83], identifying chemo-resistant [84] and immune-compromised [85] subgroups.

### 3.2. Extracellular Matrix and the Interaction with the Immune System

In the last decade, immunotherapy has radically changed the treatment for tumor patients. However, the major obstacle to this successful therapy seems to be the increased stiffness of tumor ECM, which limits the infiltration rate of both immunomodulatory drugs and T-lymphocytes, thus hampering contact with tumor cells [86,87]. As a result of the ECM-rich TME, the accumulation of immunosuppressive factors attracts regulatory T-cells (T-regs) and polarizes macrophages to an M2 pro-tumoral phenotype [88,89]. As positive feedback, the enrichment in T-regs and M2 macrophages strengthens the generation of an immunosuppressive microenvironment. Moreover, this condition could also be triggered by CAFs. In CCA, FAP-STAT3-CCL2 signaling has been identified in CAFs as sufficient to induce a deleterious inflammatory program by recruiting MDSCs and triggering an immunosuppressive TME [90]. Thus, the remodeling of tumoral ECM is recognized mainly as a crucial factor in controlling immune cell infiltration, differentiation, activation, and polarization [91,92].

Normalization of the TME can be reached by degrading tumor ECM. In this context, using collagenase in a pre-clinical lung cancer model led to the disruption of aligned collagen fibers surrounding the tumor stroma and enhanced T-cell migration [93]. A similar observation was made in PDAC, where using the PEGH20 hyaluronidase generated small windows inside the compacted matrix, increasing the efficacy of coupled chemotherapy [17]. In CCA, the disruption of the collagen matrix was complementary to the inhibition of collagen fibers cross-linking. The less dense and structured ECM boosted the infiltration and motility of T-cells, increasing the contact between tumor and T-cells, and improving the efficacy of anti-PD-1 immunotherapy [25].

Apart from biochemical disruption of tumor ECM, physical disruption of TME can be reached by localized and controlled nano-hyperthermia. Thus, a biophysical approach to induce the remodeling of tumor stroma may also promote immune surveillance and improve the success of immunotherapies. In a preclinical model of CCA, the time-limited light (photothermal therapy) or electromagnetic (magnetothermal therapy) activation of intratumorally implanted biodegradable gold-iron oxide nanoflowers (GIONFs) nanoparticles showed a significant reduction in tumor stiffness, resulting in CAF depletion and substantial ECM remodeling [94].

**Table 2:** Summary of ECM/CAFs pre-clinical and clinical trials in different tumors.

Tumor	Target	Drug	Clinical Trial	Outcomes	Ref.
CCA, PDAC, BC	Collagen	BAPN + anti-PD-1	Pre-clinical	Reduction of tumor stiffness; Remodeling of ECM; Reduction of tumor size; Increased CD8^+^ T-cell infiltration	[27]
CCA	CAFs	Navitoclax	Pre-clinical	Depletion of CAFs; Reduction of tumor size; Remodeling of ECM	[58]
CCA	CAFs αSMA^+^, ECM	PTT	Pre-clinical	Depletion of CAFs; Reduction of tumor stiffness; Remodeling of ECM; Reduction of tumor size	[91]
PDAC	HA	PEGPH20 + Gemcitabine	Pre-clinical	Depletion of HA; Inhibition of tumor growth; Improved overall survival	[17]
PDAC	CAFs αSMA^+^	Depletion + anti-CTLA-4	Pre-clinical	Depletion of CAFs combined with anti-CTLA-4; Reduction of fibrosis; Improved overall survival	[59]
PDAC	CAFs FAP^+^	Depletion/anti-CXCR4 + anti-CTLA-4/anti-PD-L1	Pre-clinical	Depletion of CAFs/CXCR4 combined with anti-CTLA-4/anti-PD-L1; Reduction of tumor size	[60]
PDAC	HA	PEGPH20 + Nab-Paclitaxel/Gemcitabine	Phase II	Depletion of HA; Inhibition of tumor growth; Improved overall survival	[80]
PDAC	HA	PEGPH20 + Nab-Paclitaxel/Gemcitabine	Phase III	Depletion of HA; No effects on overall survival; No effects on progression-free survival	[81]
LAPC	CAFs, Collagen	Losartan + Folfirinox	Phase II	Improved overall survival	[76]
CRC	CAFs FAP^+^	Sibrotuzumab	Phase II	No significant remission	[26]
OC	ECM	Losartan	Pre-clinical	Decreased ECM content	[75]
BC	CAFs, Collagen	Losartan + Dox-L	Pre-clinical	Depletion of CAFs; Reduction of Collagen I; Inhibition of tumor growth	[76]
LC	Collagen	Collagenase	Pre-clinical	Increased CD8^+^ T-cell infiltration	[90]
BC, LC, PC	CAFs FAP^+^	DNA vaccine	Pre-clinical	Depletion of FAP^+^ cells; Reduction of tumor size; Improved overall survival; No effects on prostate cancer	[61]

## 4. Conclusions

In the last decade, the new high-throughput approaches have boosted a massive integration of information deriving from different fields, enhancing the knowledge of TME and stromal remodeling at several levels.

In the context of TME, the balance between secretion and degradation of ECM is a central component contributing to the physical, mechanical, biochemical, and cellular features of the tumor. ECM composition and architecture evolve together along with tumor progression. As the main characters of the stroma, CAFs are the designers and the engineers of tumor remodeling, promoting tumor growth, regulating the progression of the disease, and affecting the therapeutic response.

The complex and diverse ECM is a scaffold with which each cellular component (CAFs, immune, tumor cells) can interact by specific cell surface receptors, impacting or being impacted within the interplay of the TME. Moreover, specific ECM and CAF signatures can have opposing functions in different tumors and stages of tumor progression, indicating that these signatures cannot be generalized to all tumor types.

As mentioned above, ECM strongly impacts tumor therapeutic resistance, trapping drugs and hampering T-cell infiltration. Therefore, the efficacy of several ECM-targeted approaches is already under evaluation, even if the dual role of ECM as pro- and anti-tumor complicates the development of ECM-targeted therapies. The recent trials involving ECM or CAF depletion compromised and worsened patient outcomes. Consequently, a better understanding of the interplay and modulation dynamics among CAFs, ECM, and immune cells could favor the development of new strategies for controlling tumoral disease, combination CAFs or ECM targeting approaches with chemotherapy or immunotherapy. Overall, these therapeutical interventions could even boost drug delivery and T-cell infiltration in desmoplastic tumors, improving the beneficial effects and patient survival.

## Figures and Tables

**Table 1:** Summary of CAF subsets and their markers found in different tumors.

Tumor	CAF Subset	Markers	Ref.
CCA	myCAF	SerpinF1^+^; POSTN^+^; VCAN^+^; Col15A1^+^; HAS2^+^	[33]
	iCAF	CXCL12^+^; HGF^+^; IL6^+^	
	mesCAF	MSLN^+^; KRT19^+^; UPK1B^+^	
CCA	myCAF	αSMA^+^; PDGFRβ^+^; FN1^+^; POSTN^+^	[44]
	iCAF	αSMA^+^; PDGFRβ^+^; Saa1^+^; FBLB1^+^	
	apCAF	αSMA^+^; PDGFRβ^+^; CD74^+^; MHCII^+^; CXCL12^+^	
	vCAF	αSMA^+^; PDGFRβ^+^; IL-6^+^; CD146^+^	
PDAC	myCAF	FAP^+^ αSMA^hi^; IL-6^low^	[32]
	iCAF	FAP^+^ αSMA^low^; IL-6^hi^; PDGFRβ^+^	
PDAC	myCAF	PDGFRα^low^; αSMA^+^; CD90^+^	[40]
	iCAF	PDGFRα^hi^; HAS1^+^; CXCL12^+^; IL-6^+^; CCL2^+^; Ly6C^+^	
	apCAF	CD74^+^; Saa3^+^; MHCII^+^	
BC	CAF-S1	CD29^med^; FAP^hi^; FSP-1^low-hi^; αSMA^hi^; PDGFRβ^med-hi^; CAV-1^low^; CCL2^+^; CCL11^+^; CXCL12^+^; CXCL14; CD73^+^; DPP4^+^	[8]
	CAF-S2	CD29^low^; FAP^neg^; FSP-1^neg-low^; αSMA^neg^; PDGFRβ^neg^; CAV-1^neg^	
	CAF-S3	CD29^med^; FAP^neg^; FSP-1^med-hi^; αSMA^neg-low^; PDGFRβ^med^; CAV-1^neg-low^	
	CAF-S4	CD29^hi^; FAP^neg-low^; FSP-1^low-med^; αSMA^hi^; PDGFRβ^low-med^; CAV-1^low^; CCL2^+^; CXCL12^+^; CXCL14^+^	
OC	CAF-S1	CD29^med-hi^; FAP^hi^; FSP-1^med-hi^; αSMA^med-hi^; PDGFRβ^med-hi^; CAV-1^low^	[41]
	CAF-S2	CD29^low^; FAP^neg^; FSP-1^neg-low^; αSMA^neg-low^; PDGFRβ^neg-low^; CAV-1^neg^	
	CAF-S3	CD29^med^; FAP^low^; FSP-1^med-hi^; αSMA^low^; PDGFRβ^med^; CAV-1^neg-low^	
	CAF-S4	CD29^hi^; FAP^low^; FSP-1^hi^; αSMA^hi^; PDGFRβ^med-hi^; CAV-1^neg-low^	

## References

[1] Ostroukhova M., Qi Z., Oriss T.B., Dixon-McCarthy B., Ray P., Ray A. (2006). Treg-mediated immunosuppression involves activation of the Notch-HES1 axis by membrane-bound TGF-beta. J. Clin. Investig..

[2] Banales J.M., Marin J.J.G., Lamarca A., Rodrigues P.M., Khan S.A., Roberts L.R., Cardinale V., Carpino G., Andersen J.B., Braconi C. (2020). Cholangiocarcinoma 2020: The next horizon in mechanisms and management. Nat. Rev. Gastroenterol. Hepatol..

[3] Khan S.A., Davidson B.R., Goldin R.D., Heaton N., Karani J., Pereira S.P., Rosenberg W.M., Tait P., Taylor-Robinson S.D., Thillainayagam A.V. (2012). Guidelines for the diagnosis and treatment of cholangiocarcinoma: An update. Gut.

[4] Mavros M.N., Economopoulos K.P., Alexiou V.G., Pawlik T.M. (2014). Treatment and Prognosis for Patients With Intrahepatic Cholangiocarcinoma: Systematic Review and Meta-analysis. JAMA Surg..

[5] Kay E.J., Paterson K., Riera-Domingo C., Sumpton D., Dabritz J.H.M., Tardito S., Boldrini C., Hernandez-Fernaud J.R., Athineos D., Dhayade S. (2022). Cancer-associated fibroblasts require proline synthesis by PYCR1 for the deposition of pro-tumorigenic extracellular matrix. Nat. Metab..

[6] Chakravarthy A., Khan L., Bensler N.P., Bose P., De Carvalho D.D. (2018). TGF-beta-associated extracellular matrix genes link cancer-associated fibroblasts to immune evasion and immunotherapy failure. Nat. Commun..

[7] Sun Y., Campisi J., Higano C., Beer T.M., Porter P., Coleman I., True L., Nelson P.S. (2012). Treatment-induced damage to the tumor microenvironment promotes prostate cancer therapy resistance through WNT16B. Nat. Med..

[8] Costa A., Kieffer Y., Scholer-Dahirel A., Pelon F., Bourachot B., Cardon M., Sirven P., Magagna I., Fuhrmann L., Bernard C. (2018). Fibroblast Heterogeneity and Immunosuppressive Environment in Human Breast Cancer. Cancer Cell.

[9] Ligorio M., Sil S., Malagon-Lopez J., Nieman L.T., Misale S., Di Pilato M., Ebright R.Y., Karabacak M.N., Kulkarni A.S., Liu A. (2019). Stromal Microenvironment Shapes the Intratumoral Architecture of Pancreatic Cancer. Cell.

[10] Vennin C., Melenec P., Rouet R., Nobis M., Cazet A.S., Murphy K.J., Herrmann D., Reed D.A., Lucas M.C., Warren S.C. (2019). CAF hierarchy driven by pancreatic cancer cell p53-status creates a pro-metastatic and chemoresistant environment via perlecan. Nat. Commun..

[11] Vaquero J., Lobe C., Tahraoui S., Claperon A., Mergey M., Merabtene F., Wendum D., Coulouarn C., Housset C., Desbois-Mouthon C. (2018). The IGF2/IR/IGF1R Pathway in Tumor Cells and Myofibroblasts Mediates Resistance to EGFR Inhibition in Cholangiocarcinoma. Clin. Cancer Res..

[12] Kalluri R., Zeisberg M. (2006). Fibroblasts in cancer. Nat. Rev. Cancer.

[13] Ohlund D., Elyada E., Tuveson D. (2014). Fibroblast heterogeneity in the cancer wound. J. Exp. Med..

[14] Banales J.M., Cardinale V., Carpino G., Marzioni M., Andersen J.B., Invernizzi P., Lind G.E., Folseraas T., Forbes S.J., Fouassier L. (2016). Expert consensus document: Cholangiocarcinoma: Current knowledge and future perspectives consensus statement from the European Network for the Study of Cholangiocarcinoma (ENS-CCA). Nat. Rev. Gastroenterol. Hepatol..

[15] Itano N., Atsumi F., Sawai T., Yamada Y., Miyaishi O., Senga T., Hamaguchi M., Kimata K. (2002). Abnormal accumulation of hyaluronan matrix diminishes contact inhibition of cell growth and promotes cell migration. Proc. Natl. Acad. Sci. USA.

[16] Sun X., Wu B., Chiang H.C., Deng H., Zhang X., Xiong W., Liu J., Rozeboom A.M., Harris B.T., Blommaert E. (2021). Tumour DDR1 promotes collagen fibre alignment to instigate immune exclusion. Nature.

[17] Jacobetz M.A., Chan D.S., Neesse A., Bapiro T.E., Cook N., Frese K.K., Feig C., Nakagawa T., Caldwell M.E., Zecchini H.I. (2013). Hyaluronan impairs vascular function and drug delivery in a mouse model of pancreatic cancer. Gut.

[18] Laklai H., Miroshnikova Y.A., Pickup M.W., Collisson E.A., Kim G.E., Barrett A.S., Hill R.C., Lakins J.N., Schlaepfer D.D., Mouw J.K. (2016). Genotype tunes pancreatic ductal adenocarcinoma tissue tension to induce matricellular fibrosis and tumor progression. Nat. Med..

[19] Olivares O., Mayers J.R., Gouirand V., Torrence M.E., Gicquel T., Borge L., Lac S., Roques J., Lavaut M.N., Berthezene P. (2017). Collagen-derived proline promotes pancreatic ductal adenocarcinoma cell survival under nutrient limited conditions. Nat. Commun..

[20] Pankova D., Chen Y., Terajima M., Schliekelman M.J., Baird B.N., Fahrenholtz M., Sun L., Gill B.J., Vadakkan T.J., Kim M.P. (2016). Cancer-Associated Fibroblasts Induce a Collagen Cross-link Switch in Tumor Stroma. Mol. Cancer Res..

[21] Veenstra V.L., Garcia-Garijo A., van Laarhoven H.W., Bijlsma M.F. (2018). Extracellular Influences: Molecular Subclasses and the Microenvironment in Pancreatic Cancer. Cancers.

[22] Bolm L., Cigolla S., Wittel U.A., Hopt U.T., Keck T., Rades D., Bronsert P., Wellner U.F. (2017). The Role of Fibroblasts in Pancreatic Cancer: Extracellular Matrix Versus Paracrine Factors. Transl. Oncol..

[23] van Tienderen G.S., Rosmark O., Lieshout R., Willemse J., de Weijer F., Elowsson Rendin L., Westergren-Thorsson G., Doukas M., Groot Koerkamp B., van Royen M.E. (2023). Extracellular matrix drives tumor organoids toward desmoplastic matrix deposition and mesenchymal transition. Acta Biomater..

[24] Hofheinz R.D., al-Batran S.E., Hartmann F., Hartung G., Jager D., Renner C., Tanswell P., Kunz U., Amelsberg A., Kuthan H. (2003). Stromal antigen targeting by a humanised monoclonal antibody: An early phase II trial of sibrotuzumab in patients with metastatic colorectal cancer. Onkologie.

[25] Nicolas-Boluda A., Vaquero J., Vimeux L., Guilbert T., Barrin S., Kantari-Mimoun C., Ponzo M., Renault G., Deptula P., Pogoda K. (2021). Tumor stiffening reversion through collagen crosslinking inhibition improves T cell migration and anti-PD-1 treatment. Elife.

[26] Guibal A., Boularan C., Bruce M., Vallin M., Pilleul F., Walter T., Scoazec J.Y., Boublay N., Dumortier J., Lefort T. (2013). Evaluation of shearwave elastography for the characterisation of focal liver lesions on ultrasound. Eur. Radiol..

[27] Masuzaki R., Tateishi R., Yoshida H., Sato T., Ohki T., Goto T., Yoshida H., Sato S., Sugioka Y., Ikeda H. (2007). Assessing liver tumor stiffness by transient elastography. Hepatol. Int..

[28] Carpino G., Overi D., Melandro F., Grimaldi A., Cardinale V., Di Matteo S., Mennini G., Rossi M., Alvaro D., Barnaba V. (2019). Matrisome analysis of intrahepatic cholangiocarcinoma unveils a peculiar cancer-associated extracellular matrix structure. Clin. Proteom..

[29] Guedj N., Blaise L., Cauchy F., Albuquerque M., Soubrane O., Paradis V. (2021). Prognostic value of desmoplastic stroma in intrahepatic cholangiocarcinoma. Mod. Pathol..

[30] Conklin M.W., Eickhoff J.C., Riching K.M., Pehlke C.A., Eliceiri K.W., Provenzano P.P., Friedl A., Keely P.J. (2011). Aligned collagen is a prognostic signature for survival in human breast carcinoma. Am. J. Pathol..

[31] Liao Z., Tan Z.W., Zhu P., Tan N.S. (2019). Cancer-associated fibroblasts in tumor microenvironment-Accomplices in tumor malignancy. Cell Immunol..

[32] Ohlund D., Handly-Santana A., Biffi G., Elyada E., Almeida A.S., Ponz-Sarvise M., Corbo V., Oni T.E., Hearn S.A., Lee E.J. (2017). Distinct populations of inflammatory fibroblasts and myofibroblasts in pancreatic cancer. J. Exp. Med..

[33] Affo S., Nair A., Brundu F., Ravichandra A., Bhattacharjee S., Matsuda M., Chin L., Filliol A., Wen W., Song X. (2021). Promotion of cholangiocarcinoma growth by diverse cancer-associated fibroblast subpopulations. Cancer Cell.

[34] Sahai E., Astsaturov I., Cukierman E., DeNardo D.G., Egeblad M., Evans R.M., Fearon D., Greten F.R., Hingorani S.R., Hunter T. (2020). A framework for advancing our understanding of cancer-associated fibroblasts. Nat. Rev. Cancer.

[35] Ao Z., Shah S.H., Machlin L.M., Parajuli R., Miller P.C., Rawal S., Williams A.J., Cote R.J., Lippman M.E., Datar R.H. (2015). Identification of Cancer-Associated Fibroblasts in Circulating Blood from Patients with Metastatic Breast Cancer. Cancer Res..

[36] Moffitt R.A., Marayati R., Flate E.L., Volmar K.E., Loeza S.G., Hoadley K.A., Rashid N.U., Williams L.A., Eaton S.C., Chung A.H. (2015). Virtual microdissection identifies distinct tumor- and stroma-specific subtypes of pancreatic ductal adenocarcinoma. Nat. Genet..

[37] Neuzillet C., Tijeras-Raballand A., Ragulan C., Cros J., Patil Y., Martinet M., Erkan M., Kleeff J., Wilson J., Apte M. (2019). Inter- and intra-tumoural heterogeneity in cancer-associated fibroblasts of human pancreatic ductal adenocarcinoma. J. Pathol..

[38] Lambrechts D., Wauters E., Boeckx B., Aibar S., Nittner D., Burton O., Bassez A., Decaluwe H., Pircher A., Van den Eynde K. (2018). Phenotype molding of stromal cells in the lung tumor microenvironment. Nat. Med..

[39] Biffi G., Oni T.E., Spielman B., Hao Y., Elyada E., Park Y., Preall J., Tuveson D.A. (2019). IL1-Induced JAK/STAT Signaling Is Antagonized by TGFbeta to Shape CAF Heterogeneity in Pancreatic Ductal Adenocarcinoma. Cancer Discov..

[40] Elyada E., Bolisetty M., Laise P., Flynn W.F., Courtois E.T., Burkhart R.A., Teinor J.A., Belleau P., Biffi G., Lucito M.S. (2019). Cross-Species Single-Cell Analysis of Pancreatic Ductal Adenocarcinoma Reveals Antigen-Presenting Cancer-Associated Fibroblasts. Cancer Discov..

[41] Givel A.M., Kieffer Y., Scholer-Dahirel A., Sirven P., Cardon M., Pelon F., Magagna I., Gentric G., Costa A., Bonneau C. (2018). miR200-regulated CXCL12beta promotes fibroblast heterogeneity and immunosuppression in ovarian cancers. Nat. Commun..

[42] Kieffer Y., Hocine H.R., Gentric G., Pelon F., Bernard C., Bourachot B., Lameiras S., Albergante L., Bonneau C., Guyard A. (2020). Single-Cell Analysis Reveals Fibroblast Clusters Linked to Immunotherapy Resistance in Cancer. Cancer Discov..

[43] Li H., Courtois E.T., Sengupta D., Tan Y., Chen K.H., Goh J.J.L., Kong S.L., Chua C., Hon L.K., Tan W.S. (2017). Reference component analysis of single-cell transcriptomes elucidates cellular heterogeneity in human colorectal tumors. Nat. Genet..

[44] Zhang M., Yang H., Wan L., Wang Z., Wang H., Ge C., Liu Y., Hao Y., Zhang D., Shi G. (2020). Single-cell transcriptomic architecture and intercellular crosstalk of human intrahepatic cholangiocarcinoma. J. Hepatol..

[45] Job S., Rapoud D., Dos Santos A., Gonzalez P., Desterke C., Pascal G., Elarouci N., Ayadi M., Adam R., Azoulay D. (2020). Identification of Four Immune Subtypes Characterized by Distinct Composition and Functions of Tumor Microenvironment in Intrahepatic Cholangiocarcinoma. Hepatology.

[46] Martin-Serrano M.A., Kepecs B., Torres-Martin M., Bramel E.R., Haber P.K., Merritt E., Rialdi A., Param N.J., Maeda M., Lindblad K.E. (2023). Novel microenvironment-based classification of intrahepatic cholangiocarcinoma with therapeutic implications. Gut.

[47] Aizawa T., Karasawa H., Funayama R., Shirota M., Suzuki T., Maeda S., Suzuki H., Yamamura A., Naitoh T., Nakayama K. (2019). Cancer-associated fibroblasts secrete Wnt2 to promote cancer progression in colorectal cancer. Cancer Med..

[48] Linares J., Marin-Jimenez J.A., Badia-Ramentol J., Calon A. (2020). Determinants and Functions of CAFs Secretome During Cancer Progression and Therapy. Front. Cell Dev. Biol..

[49] Li Z., Zhang J., Zhou J., Lu L., Wang H., Zhang G., Wan G., Cai S., Du J. (2019). Nodal Facilitates Differentiation of Fibroblasts to Cancer-Associated Fibroblasts that Support Tumor Growth in Melanoma and Colorectal Cancer. Cells.

[50] Kalluri R. (2016). The biology and function of fibroblasts in cancer. Nat. Rev. Cancer.

[51] Cangkrama M., Wietecha M., Mathis N., Okumura R., Ferrarese L., Al-Nuaimi D., Antsiferova M., Dummer R., Innocenti M., Werner S. (2020). A paracrine activin A-mDia2 axis promotes squamous carcinogenesis via fibroblast reprogramming. EMBO Mol. Med..

[52] Senthebane D.A., Rowe A., Thomford N.E., Shipanga H., Munro D., Mazeedi M., Almazyadi H.A.M., Kallmeyer K., Dandara C., Pepper M.S. (2017). The Role of Tumor Microenvironment in Chemoresistance: To Survive, Keep Your Enemies Closer. Int. J. Mol. Sci..

[53] Liu T., Zhou L., Li D., Andl T., Zhang Y. (2019). Cancer-Associated Fibroblasts Build and Secure the Tumor Microenvironment. Front. Cell Dev. Biol..

[54] Huang T.X., Tan X.Y., Huang H.S., Li Y.T., Liu B.L., Liu K.S., Chen X., Chen Z., Guan X.Y., Zou C. (2022). Targeting cancer-associated fibroblast-secreted WNT2 restores dendritic cell-mediated antitumour immunity. Gut.

[55] Wang M., Jiang H., Liu X., Wang X. (2022). Biophysics involved in the process of tumor immune escape. iScience.

[56] Du H., Bartleson J.M., Butenko S., Alonso V., Liu W.F., Winer D.A., Butte M.J. (2023). Tuning immunity through tissue mechanotransduction. Nat. Rev. Immunol..

[57] Mertens J.C., Fingas C.D., Christensen J.D., Smoot R.L., Bronk S.F., Werneburg N.W., Gustafson M.P., Dietz A.B., Roberts L.R., Sirica A.E. (2013). Therapeutic effects of deleting cancer-associated fibroblasts in cholangiocarcinoma. Cancer Res..

[58] Feig C., Jones J.O., Kraman M., Wells R.J., Deonarine A., Chan D.S., Connell C.M., Roberts E.W., Zhao Q., Caballero O.L. (2013). Targeting CXCL12 from FAP-expressing carcinoma-associated fibroblasts synergizes with anti-PD-L1 immunotherapy in pancreatic cancer. Proc. Natl. Acad. Sci. USA.

[59] Ozdemir B.C., Pentcheva-Hoang T., Carstens J.L., Zheng X., Wu C.C., Simpson T.R., Laklai H., Sugimoto H., Kahlert C., Novitskiy S.V. (2014). Depletion of carcinoma-associated fibroblasts and fibrosis induces immunosuppression and accelerates pancreas cancer with reduced survival. Cancer Cell.

[60] Duperret E.K., Trautz A., Ammons D., Perales-Puchalt A., Wise M.C., Yan J., Reed C., Weiner D.B. (2018). Alteration of the Tumor Stroma Using a Consensus DNA Vaccine Targeting Fibroblast Activation Protein (FAP) Synergizes with Antitumor Vaccine Therapy in Mice. Clin. Cancer Res..

[61] Dominguez C.X., Muller S., Keerthivasan S., Koeppen H., Hung J., Gierke S., Breart B., Foreman O., Bainbridge T.W., Castiglioni A. (2020). Single-Cell RNA Sequencing Reveals Stromal Evolution into LRRC15(+) Myofibroblasts as a Determinant of Patient Response to Cancer Immunotherapy. Cancer Discov..

[62] Kemi N., Eskuri M., Herva A., Leppanen J., Huhta H., Helminen O., Saarnio J., Karttunen T.J., Kauppila J.H. (2018). Tumour-stroma ratio and prognosis in gastric adenocarcinoma. Br. J. Cancer.

[63] Graizel D., Zlotogorski-Hurvitz A., Tsesis I., Rosen E., Kedem R., Vered M. (2020). Oral cancer-associated fibroblasts predict poor survival: Systematic review and meta-analysis. Oral Dis..

[64] Provenzano P.P., Inman D.R., Eliceiri K.W., Knittel J.G., Yan L., Rueden C.T., White J.G., Keely P.J. (2008). Collagen density promotes mammary tumor initiation and progression. BMC Med..

[65] Lu P., Weaver V.M., Werb Z. (2012). The extracellular matrix: A dynamic niche in cancer progression. J. Cell Biol..

[66] Levental K.R., Yu H., Kass L., Lakins J.N., Egeblad M., Erler J.T., Fong S.F., Csiszar K., Giaccia A., Weninger W. (2009). Matrix crosslinking forces tumor progression by enhancing integrin signaling. Cell.

[67] Taufalele P.V., VanderBurgh J.A., Munoz A., Zanotelli M.R., Reinhart-King C.A. (2019). Fiber alignment drives changes in architectural and mechanical features in collagen matrices. PLoS ONE.

[68] Provenzano P.P., Eliceiri K.W., Campbell J.M., Inman D.R., White J.G., Keely P.J. (2006). Collagen reorganization at the tumor-stromal interface facilitates local invasion. BMC Med..

[69] Provenzano P.P., Inman D.R., Eliceiri K.W., Trier S.M., Keely P.J. (2008). Contact guidance mediated three-dimensional cell migration is regulated by Rho/ROCK-dependent matrix reorganization. Biophys. J..

[70] Han W., Chen S., Yuan W., Fan Q., Tian J., Wang X., Chen L., Zhang X., Wei W., Liu R. (2016). Oriented collagen fibers direct tumor cell intravasation. Proc. Natl. Acad. Sci. USA.

[71] Ajeti V., Nadiarnykh O., Ponik S.M., Keely P.J., Eliceiri K.W., Campagnola P.J. (2011). Structural changes in mixed Col I/Col V collagen gels probed by SHG microscopy: Implications for probing stromal alterations in human breast cancer. Biomed Opt. Express.

[72] Bar J.K., Grelewski P., Popiela A., Noga L., Rabczynski J. (2004). Type IV collagen and CD44v6 expression in benign, malignant primary and metastatic ovarian tumors: Correlation with Ki-67 and p53 immunoreactivity. Gynecol. Oncol..

[73] Fang S., Dai Y., Mei Y., Yang M., Hu L., Yang H., Guan X., Li J. (2019). Clinical significance and biological role of cancer-derived Type I collagen in lung and esophageal cancers. Thorac. Cancer.

[74] Miskolczi Z., Smith M.P., Rowling E.J., Ferguson J., Barriuso J., Wellbrock C. (2018). Collagen abundance controls melanoma phenotypes through lineage-specific microenvironment sensing. Oncogene.

[75] Zhao Y., Cao J., Melamed A., Worley M., Gockley A., Jones D., Nia H.T., Zhang Y., Stylianopoulos T., Kumar A.S. (2019). Losartan treatment enhances chemotherapy efficacy and reduces ascites in ovarian cancer models by normalizing the tumor stroma. Proc. Natl. Acad. Sci. USA.

[76] Hu C., Liu X., Ran W., Meng J., Zhai Y., Zhang P., Yin Q., Yu H., Zhang Z., Li Y. (2017). Regulating cancer associated fibroblasts with losartan-loaded injectable peptide hydrogel to potentiate chemotherapy in inhibiting growth and lung metastasis of triple negative breast cancer. Biomaterials.

[77] Murphy J.E., Wo J.Y., Ryan D.P., Clark J.W., Jiang W., Yeap B.Y., Drapek L.C., Ly L., Baglini C.V., Blaszkowsky L.S. (2019). Total Neoadjuvant Therapy With FOLFIRINOX in Combination With Losartan Followed by Chemoradiotherapy for Locally Advanced Pancreatic Cancer: A Phase 2 Clinical Trial. JAMA Oncol..

[78] Cheng X.B., Sato N., Kohi S., Yamaguchi K. (2013). Prognostic impact of hyaluronan and its regulators in pancreatic ductal adenocarcinoma. PLoS ONE.

[79] Lipponen P., Aaltomaa S., Tammi R., Tammi M., Agren U., Kosma V.M. (2001). High stromal hyaluronan level is associated with poor differentiation and metastasis in prostate cancer. Eur. J. Cancer.

[80] Hingorani S.R., Zheng L., Bullock A.J., Seery T.E., Harris W.P., Sigal D.S., Braiteh F., Ritch P.S., Zalupski M.M., Bahary N. (2018). HALO 202: Randomized Phase II Study of PEGPH20 Plus Nab-Paclitaxel/Gemcitabine Versus Nab-Paclitaxel/Gemcitabine in Patients With Untreated, Metastatic Pancreatic Ductal Adenocarcinoma. J. Clin. Oncol..

[81] Van Cutsem E., Tempero M.A., Sigal D., Oh D.-Y., Fazio N., Macarulla T., Hitre E., Hammel P., Hendifar A.E., Bates S.E. (2020). Randomized Phase III Trial of Pegvorhyaluronidase Alfa With Nab-Paclitaxel Plus Gemcitabine for Patients With Hyaluronan-High Metastatic Pancreatic Adenocarcinoma. J. Clin. Oncol..

[82] Jiang K., Liu H., Xie D., Xiao Q. (2019). Differentially expressed genes ASPN, COL1A1, FN1, VCAN and MUC5AC are potential prognostic biomarkers for gastric cancer. Oncol. Lett..

[83] Bergamaschi A., Tagliabue E., Sorlie T., Naume B., Triulzi T., Orlandi R., Russnes H.G., Nesland J.M., Tammi R., Auvinen P. (2008). Extracellular matrix signature identifies breast cancer subgroups with different clinical outcome. J. Pathol..

[84] Mintz M.B., Sowers R., Brown K.M., Hilmer S.C., Mazza B., Huvos A.G., Meyers P.A., Lafleur B., McDonough W.S., Henry M.M. (2005). An expression signature classifies chemotherapy-resistant pediatric osteosarcoma. Cancer Res..

[85] Chen D., Chen D., Cao D., Hu J., Yao Y. (2018). A signature based on survival-related genes identifies high-risk glioblastomas harboring immunosuppressive and aggressive ECM characteristics. Zhong Nan Da Xue Xue Bao Yi Xue Ban.

[86] Raave R., van Kuppevelt T.H., Daamen W.F. (2018). Chemotherapeutic drug delivery by tumoral extracellular matrix targeting. J. Control Release.

[87] Issa-Nummer Y., Darb-Esfahani S., Loibl S., Kunz G., Nekljudova V., Schrader I., Sinn B.V., Ulmer H.U., Kronenwett R., Just M. (2013). Prospective validation of immunological infiltrate for prediction of response to neoadjuvant chemotherapy in HER2-negative breast cancer--a substudy of the neoadjuvant GeparQuinto trial. PLoS ONE.

[88] Ruella M., Klichinsky M., Kenderian S.S., Shestova O., Ziober A., Kraft D.O., Feldman M., Wasik M.A., June C.H., Gill S. (2017). Overcoming the Immunosuppressive Tumor Microenvironment of Hodgkin Lymphoma Using Chimeric Antigen Receptor T Cells. Cancer Discov..

[89] Kim H., Cha J., Jang M., Kim P. (2019). Hyaluronic acid-based extracellular matrix triggers spontaneous M2-like polarity of monocyte/macrophage. Biomater. Sci..

[90] Yang X., Lin Y., Shi Y., Li B., Liu W., Yin W., Dang Y., Chu Y., Fan J., He R. (2016). FAP Promotes Immunosuppression by Cancer-Associated Fibroblasts in the Tumor Microenvironment via STAT3-CCL2 Signaling. Cancer Res..

[91] Vaday G.G., Lider O. (2000). Extracellular matrix moieties, cytokines, and enzymes: Dynamic effects on immune cell behavior and inflammation. J. Leukoc. Biol..

[92] Mushtaq M.U., Papadas A., Pagenkopf A., Flietner E., Morrow Z., Chaudhary S.G., Asimakopoulos F. (2018). Tumor matrix remodeling and novel immunotherapies: The promise of matrix-derived immune biomarkers. J. Immunother. Cancer.

[93] Salmon H., Franciszkiewicz K., Damotte D., Dieu-Nosjean M.C., Validire P., Trautmann A., Mami-Chouaib F., Donnadieu E. (2012). Matrix architecture defines the preferential localization and migration of T cells into the stroma of human lung tumors. J. Clin. Investig.

[94] Nicolas-Boluda A., Vaquero J., Laurent G., Renault G., Bazzi R., Donnadieu E., Roux S., Fouassier L., Gazeau F. (2020). Photothermal Depletion of Cancer-Associated Fibroblasts Normalizes Tumor Stiffness in Desmoplastic Cholangiocarcinoma. ACS Nano.

